# Efficient Assay and Marker Significance of NAD^+^ in Human Blood

**DOI:** 10.3389/fmed.2022.886485

**Published:** 2022-05-19

**Authors:** Natalia V. Balashova, Lev G. Zavileyskiy, Artem V. Artiukhov, Leonid A. Shaposhnikov, Olga P. Sidorova, Vladimir I. Tishkov, Angela Tramonti, Anastasia A. Pometun, Victoria I. Bunik

**Affiliations:** ^1^Department of Clinical Laboratory Diagnostics, Faculty of Advanced Medicine, M.F. Vladimirsky Moscow Regional Research and Clinical Institute (MONIKI), Moscow, Russia; ^2^Department of Dietetics and Clinical Nutritionology, Faculty of Continuing Medical Education, RUDN Medical Institute, Moscow, Russia; ^3^Faculty of Bioengineering and Bioinformatics, Lomonosov Moscow State University, Moscow, Russia; ^4^Department of Biokinetics, A. N. Belozersky Institute of Physico-Chemical Biology, Lomonosov Moscow State University, Moscow, Russia; ^5^Department of Biochemistry, Sechenov University, Moscow, Russia; ^6^Department of Chemical Enzymology, Faculty of Chemistry, Lomonosov Moscow State University, Moscow, Russia; ^7^Department of Neurology, Faculty of Advanced Medicine, M.F. Vladimirsky Moscow Regional Research and Clinical Institute (MONIKI), Moscow, Russia; ^8^Bach Institute of Biochemistry, Federal Research Centre “Fundamentals of Biotechnology” of the Russian Academy of Sciences, Moscow, Russia; ^9^Institute of Molecular Biology and Pathology, Italian National Research Council, Department of Biochemical Sciences “A. Rossi Fanelli,” Sapienza University of Rome, Rome, Italy

**Keywords:** NAD^+^ in human blood, formate dehydrogenase, cardiac patient, neurological patient, metabolic markers, Charcot-Marie-Tooth disease

## Abstract

Oxidized nicotinamide adenine dinucleotide (NAD^+^) is a biological molecule of systemic importance. Essential role of NAD^+^ in cellular metabolism relies on the substrate action in various redox reactions and cellular signaling. This work introduces an efficient enzymatic assay of NAD^+^ content in human blood using recombinant formate dehydrogenase (FDH, EC 1.2.1.2), and demonstrates its diagnostic potential, comparing NAD^+^ content in the whole blood of control subjects and patients with cardiac or neurological pathologies. In the control group (*n* = 22, 25–70 years old), our quantification of the blood concentration of NAD^+^ (18 μM, minimum 15, max 23) corresponds well to NAD^+^ quantifications reported in literature. In patients with demyelinating neurological diseases (*n* = 10, 18–55 years old), the NAD^+^ levels significantly (*p* < 0.0001) decrease (to 14 μM, min 13, max 16), compared to the control group. In cardiac patients with the heart failure of stage II and III according to the New York Heart Association (NYHA) functional classification (*n* = 24, 42–83 years old), the blood levels of NAD^+^ (13 μM, min 9, max 18) are lower than those in the control subjects (*p* < 0.0001) or neurological patients (*p* = 0.1). A better discrimination of the cardiac and neurological patients is achieved when the ratios of NAD^+^ to the blood creatinine levels, mean corpuscular volume or potassium ions are compared. The proposed NAD^+^ assay provides an easy and robust tool for clinical analyses of an important metabolic indicator in the human blood.

## Introduction

Nicotinamide adenine dinucleotide (NAD, the sum of the oxidised NAD^+^ and reduced NADH forms) is a very important biological molecule which is involved in various metabolic and signaling pathways. Undergoing reversible reduction to NADH in many redox reactions, NAD^+^ is also involved in signaling of perturbed homeostasis and DNA damage response. The signaling pathways include the NAD^+^-degrading reactions catalyzed by protein deacylases sirtuins (1–3), poly(ADP-ribose) polymerases 1 and 2 involved in the DNA damage response (4, 5), and NAD^+^ hydrolyzing enzymes CD38 (6)/CD157 (7). These NAD^+^-dependent reactions are involved in regulation of circadian rhythms (8), aging (9–11), and immunity (12). Major portion of NAD^+^ resides inside the cells, where its concentration may vary from 0.01 to 1 mM, yet some studies also determine significantly lower quantities of NAD^+^ in the blood plasma [2–70 nM in humans (10, 13, 14) and 240–290 nM in pigs (15)].

The correlation between the concentrations of NAD^+^ in blood/plasma and tissues has been studied in aging (16–18). The reduction in NAD^+^ level with age is observed in healthy human brain (19, 20), liver (21), red blood cells (22) and macrophages (23). In plasma, the NAD^+^ level is shown to drop from app. 50 nM in young (20–40 years) to app. 10 nM in elderly subjects (60–87 years) (10). Human skin NAD^+^ content also sharply declines as people age: from 8.5 ± 1.6 ng/mg protein in newborns to 1.1 ± 0.2 in elders (>51 years) (9). A strong negative correlation is observed between NAD^+^ levels in skin and age in both males (*r* = −0.706; *p* = 0.001) and females (*r* = −0.537; *p* = 0.01) (9). The total pool of NAD (both NAD^+^ and NADH) in whole blood, however, shows a trend to a negative correlation with age in males that is not observed in females (11). In the population combined from both genders, blood NAD levels in elderly patients (75–101 years old) hospitalized for decompensated heart failure are shown to be lower (20.7 ± 3.6 μM) than those in a healthy population of 151 voluntary donors aged 19 to 68 years (23.4 ± 4.1 μM) (11).

Changed NAD levels may be associated with cancer (16, 24, 25), obesity and type 2 diabetes (16, 26, 27), various neurological disorders (28–30), intestinal inflammation (31). Many studies point to decreased NAD levels under disturbed nutrient conditions (26). In view of the wide range of pathologies potentially affecting the NAD levels and redox state, the quantitative determination of this metabolite in human blood may be of diagnostic value. Worth noting, a rapid and efficient assay of NAD^+^ may be extremely useful to decide on treatments of critically ill patients, as the tissue damage response is associated with the NAD^+^ depletion in the poly(ADP-ribose)- polymerase-catalyzed reaction (4, 5). As a basic indicator of the healthy metabolism, NAD^+^ level has a potential to be used as a marker of biological age or nutritional state.

Recently, we have published the method of NAD^+^ quantitative determination using recombinant formate dehydrogenase (FDH) (32), whose application to the extracts of the rat brain tissue and its mitochondria has demonstrated such advantages of the assay as its high sensitivity and specificity. Compared to the “gold-standard” HPLC- and/or mass-spectrometry-based methods, our FDH assay does not require expensive consumables, neither highly professional supervision. A number of already existing biotechnological applications of FDH (33), also as fusion protein (34–36), enzyme mixture (37, 38) or in whole cell biocatalysis (39), extending from NAD(P)H regeneration to fixing atmospheric CO_2_ (40–43), demonstrate the enzyme robustness and utility for the environmentally friendly procedures. In this regard, development of the FDH-based clinical assays has another advantage over the currently employed assays of total NAD pool, using formazan dyes, as the most employed 3-(4,5-dimethylthiazol-2-yl)-2,5-diphenyltetrazolium bromide, known as MTT, is toxic for eucaryotic cells (44, 45). In the present study, we use FDH from *Pseudomonas sp.* that is extremely specific to NAD^+^, i.e., does not catalyze the reduction of NADP^+^, and is characterized by high catalytic efficiency and high thermal stability, compared to FDH from other sources (33, 46, 47). Employing this enzyme and developing the optimized protocol of the extraction of NAD^+^ from blood, we demonstrate the diagnostic potential of the assay for medical application by measuring the whole blood NAD^+^ in the healthy subjects and patients with neurological and cardiological pathologies.

## Methods

### Enrollment of Patients in the Study

The study was approved by the ethics commission of M.F. Vladimirsky Moscow Regional Research and Clinical Institute (MONIKI), decision N 17 of 10. December, 2020. All participants gave informed consent. The neurological patients with demyelinating diseases and cardiological patients with the heart failure of stage II and III according to the New York Heart Association (NYHA) functional classification, were enrolled in the study (Table 1) during 1 year. Our choice of the NYHA heart failure stages II and III was based on clinical abundance of these cardiological patients, in contrast to those of stage I, and a lower occurrence in these patients of additional deteriorations associated with the profound pathology of stage IV.

**TABLE 1:** Summary of the studied cohorts where the NAD^+^ content in the whole blood is quantified.

Group	Sex	Age, years	Main diagnosis
Healthy controls, Total *n* = 22	45% males (*n* = 10)	25–70	None reported
Neurological patients, Total *n* = 10	40% males (*n* = 4)	18–55	Charcot-marie-tooth neuropathy (*n* = 7)
			Multiple sclerosis (*n* = 1)
			Okinawa neuropathy (*n* = 2)
Cardiological patients, Total *n* = 28	All: 79% males (*n* = 22)	42–83	Heart failure stage NYHA: II – 46% (*n* = 13), III – 54% (*n* = 15)
	Without outliers (*n* = 4, all males): 75% males (*n* = 18), Total *n* = 24	42–83	All outliers are determined in the patients with the heart failure of stage III. Heart failure stage NYHA: II – 54% (*n* = 13), III – 46% (*n* = 11)

*The outliers of the determined NAD^+^ content in a cohort are identified by the interquartile range rule using 1.5 IQR criterion.*

### Preparation of Recombinant Formate Dehydrogenase

Recombinant FDH from *Pseudomonas* sp. was produced in *E. coli* as described in (48). The enzyme (1.1 mg/mL, app. 10 U/mg) was stored in 0.1 M sodium phosphate buffer, pH 7.0, containing 10 mM EDTA, at + 4°C.

### Preparation of Methanol-Acetic Acid Extracts of Whole Blood

The blood was collected in the morning in a vacutainer tube with heparin (6 ml), aliquoted in 1 ml and frozen at −70°C. The blood levels of NAD^+^ did not decay upon the blood storage up to several months. Typically, the samples were accumulated and extracted within 2 months after the blood collection. A modification of the extraction protocol previously elaborated for the rat brain (49) was used. To prepare the extracts, blood samples were thawed on ice, 0.2 ml of each sample was transferred into a clean microcentrifuge tube and mixed with 1.6 ml of methanol precooled at + 4°C, using the T10 Basic ULTRA-TURRAX disperser (IKA, Staufen, Germany). 0.27 ml of 2% acetic acid was added, followed by 30 min shaking on ice in New Brunswick Excella E24R incubator (Eppendorf, Moscow, Russia) at 180 rpm. The resulting suspension was deproteinized by 20 min centrifugation at 21,500 *g* and 4°C, using Hitachi CT15RE centrifuge (Helicon, Moscow, Russia). The supernatant was transferred into a clean tube and stored at −70°C until analysis, usually performed the day after the extract preparations. Repeated assays of the same blood extracts before and after the storage showed that their NAD^+^ content was stable during several months.

### NAD^+^ Determination Procedure

NAD^+^ concentration in the blood extracts was determined enzymatically as described earlier (32), using fluorescence mode of BMG ClarioSTAR Plus plate reader (Helicon, Moscow, Russia). Samples of blood extracts were shaken and 0.01, 0.015 or 0.02 ml aliquots of each sample were added into a 96-well black microplate (Greiner #655076) in duplicates. The mixture of 87% methanol/0.3% acetic acid was added to the aliquots to obtain the total volume of 0.02 ml. The blank contained 0.02 ml of the methanol-acetic acid mixture only. 0.18 ml of 0.6 M sodium formate in 0.1 M sodium phosphate buffer, pH 7.0, was added to all the wells. The background fluorescence (340/475 nm) of the samples was measured during 6 min. After registering the background levels, app. 0.03 U of FDH (app. 3 μg) was added into each well, and the fluorescence change was measured for 20 min. Usually, a plateau in the fluorescence was reached within 10 min, pointing to the completion of the reaction. NAD^+^ content in each well was calculated using the calibration curve employing 0.01–0.1 nmol of NAD^+^ and 0.03 U of FDH per well. Our comparison to the calibration with added NADH showed that the calibration employing the FDH reaction, better reproduced exact conditions of the NAD^+^ fluorescence assay. Simultaneously, the calibration employing the FDH reaction served as an internal control for the linearity and functionality of the enzyme assay in the selected interval of the NAD^+^ concentrations. The NAD^+^ content in the whole blood was calculated, taking into account the added extract volume and a 10.2-fold dilution of blood upon the extraction procedure.

### Statistical Analysis

Comparisons between groups were made by one-way ANOVA with Tukey’s *post hoc* test or by Mann–Whitney’s U test in case of comparisons of two groups (*, ^**^, ^***^, ^****^ for *p* < 0.05, *p* < 0.01, *p* < 0.001, and *p* < 0.0001, respectively). Outliers were determined using the interquartile range rule (1.5 IQR criterion). Holm-Bonferroni correction was used for multiple testing adjustment. All statistical analysis and data visualization was performed using R Statistical Software (version 4.1.1; R Foundation for Statistical Computing, Vienna, Austria).

## Results

In our pilot study, concentrations of NAD^+^ in the whole blood are compared in three different cohorts: healthy controls, cardiological patients with the heart failure of the NYHA class II and III, and neurological patients with demyelinating diseases, such as Charcot-Marie-Tooth type, Okinawa type and multiple sclerosis (Table 1). All the analyzed neuropathies are characterized by impaired myelination of nerve fibers, either in the central (multiple sclerosis) or peripheral (neuropathies of Charcot-Marie-Tooth and Okinawa types) nervous systems. Analysis of the quantified values of the whole blood NAD^+^ within the studied cohorts using the interquartile range rule (1.5 IQR criterion) has revealed four outliers in the group of cardiological patients, all of them determined in the patients with heart failure of the NYHA stage III. All the blood parameters inherent in the studied cohorts are shown as medians, minimum and maximum values in Supplementary Table 1, which also presents *p*-values characterizing significance of the group differences.

**FIGURE 1:**
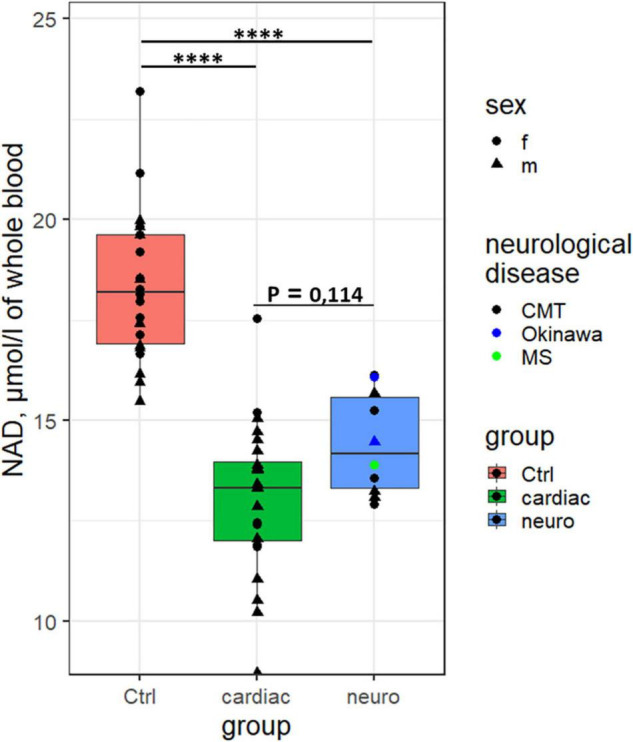
Differences in the concentration of NAD^+^ in the whole blood between the control subjects (*n* = 22), the cardiological (*n* = 24, excluding the four outliers) and neurological (*n* = 10) patients. ^****^p < 0.0001.

Independent of the exclusion (Figure 1) or inclusion (Supplementary Table 1) of the outliers, NAD^+^ concentrations in the blood of healthy subjects are significantly higher than those in the cardiological or neurological patients. No gender differences in the NAD^+^ levels are detected in our study. Cardiological patients with NYHA stage III show a trend to lower NAD^+^ levels, compared to the patients with NYHA stage II (*p* = 0.09, Supplementary Figure 1). Taking into account the known decreases in the blood NAD^+^ level with age (11) and the different median age of the studied groups (Supplementary Table 1), we have verified the finding in the cohorts using their comparison to the controls of similar age. With the studied subjects divided into the age groups of 42–68 years (cardiological patients *vs.* controls of the respective age) and 27–55 years (neurological patients *vs.* controls of respective age), significant differences in the NAD^+^ concentrations between the control subjects and cardiological or neurological patients are preserved (Supplementary Figure 2).

As the NAD^+^ levels alone cannot unambiguously discriminate the cardiological and neurological patients (*p* = 0.1, Figure 1), we have attempted to increase the discriminating power of the NAD^+^ assay by finding potentially different relationships between the NAD^+^ levels and associated blood markers in different pathologies. Correlations of the blood levels of NAD^+^ to the available parameters of the clinical and biochemical blood analyses of the studied cohorts indicate that the control correlations are always well separated from those in the patients, while the difference between the cardiological and neurological patients is not so strong (Supplementary Figure 3 and Supplementary Table 2). However, the NAD^+^ dependence on Na^+^ ions is opposite in the control subjects and neurological patients (Supplementary Figure 3 and Supplementary Table 2), complemented by statistically significant group differences between the median values of Na^+^ ions.

**FIGURE 2:**
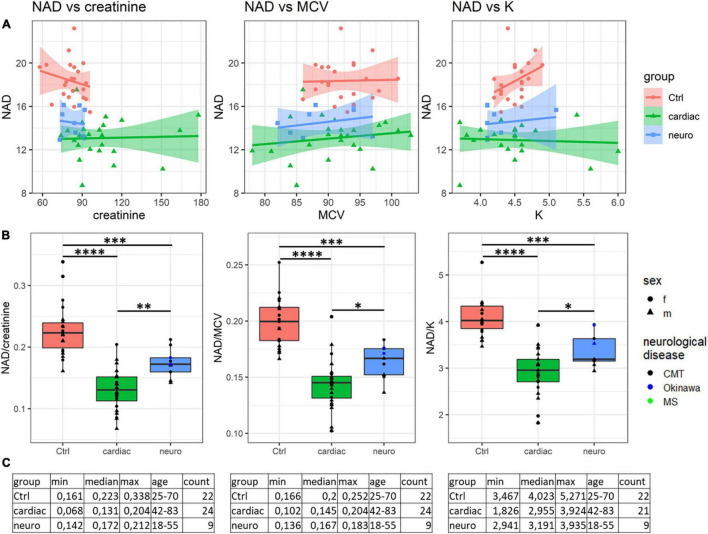
Analysis of discriminating potential of the coupled variations in the NAD^+^ concentrations and other parameters of the blood. **(A)** Correlations of the NAD^+^ concentrations with the creatinine level, mean corpuscular volume (MCV) and K^+^ ions. **(B)** Significance of the differences between the NAD^+^ ratios to creatinine, MCV and K^+^ ions, determined for each of the studied samples. **(C)** Tabular presentation of the ratios used in panel **(B)**. *p < 0.05, ^**^p < 0.01, ^***^p < 0.001, ^****^p < <0.0001.

Analysis of the ratios of NAD^+^ levels to each of the available parameters, inherent in a specific blood sample, reveals that some of the ratios may be used for a better discrimination between the neurological and cardiological patients (Table 2). Figure 2 presents such ratios for the three parameters assayed in the blood along with NAD^+^. While one of the parameters (creatinine) is characterized by statistically significant group differences in its median values, the medians of the two other parameters (MCV, mean corpuscular volume, and K^+^) do not significantly differ between the groups (Supplementary Table 1). It is worth noting in this regard that the ratios presented in Figure 2 are determined in each patient. Therefore, they manifest the coupled changes better than the overall median values of the parameters. This is exemplified in Figure 2 by the statistically significant differences between the cardiological and neurological patients in their NAD^+^ ratios to creatinine, MCV and K^+^ ions. These ratios show a higher statistical significance of the differences between the neurological and cardiological patients, than NAD^+^ levels alone (Figures 1, 2). Thus, in addition to the blood levels of NAD^+^, taking into account the coupled changes in other parameters may increase the diagnostic power of the NAD^+^ assays.

**TABLE 2:** Discriminating power of the ratios of NAD^+^ content to other blood parameters.

Ratios	*p*	*p* adj
NAD^+^/Creatinine	0.0008	0.015
NAD^+^/MCV	0.006	0.115
NAD^+^/K^+^	0.01	0.18
NAD^+^/MCH	0.01	0.18
NAD^+^/Glucose	0.01	0.18
NAD^+^/Urea	0.03	0.37
NAD^+^/Triglycerides	0.03	0.37
NAD^+^/MCHC	0.03	0.43
NAD^+^/ Na^+^	0.05	0.52
NAD^+^/Hemoglobin	0.05	0.52
NAD^+^/Hematocryt	0.05	0.52
NAD^+^/Sedimentation rate	0.05	0.52
NAD^+^/Cholesterol_total	0.06	0.52
NAD^+^/Bilirubin_total	0.08	0.52
NAD^+^/RBC	0.19	1
NAD^+^/Asp aminotransferase	0.36	1
NAD^+^/Total_protein	0.40	1
NAD^+^/Lympocytes	0.68	1
NAD^+^/Ala aminotransferase	0.74	1

*Statistical significance of the differences in the ratios inherent in the cardiological and neurological patients is analyzed by Mann–Whitney U test (p), followed by the p-values adjusted for the multiple comparison employing the Holm–Bonferroni correction.*

## Discussion

The NAD^+^ values determined in our study for the healthy volunteers are in good accordance with those of independent studies, where NAD^+^ in human blood is measured by mass spectrometry or NMR (Supplementary Table 3). Other enzymatic assays of NAD^+^ are known to employ lactate dehydrogenase or alcohol dehydrogenase. Unlike our FDH-based assay, these reactions are reversible. The reversibility makes such tests prone to conditional problems when established equilibria interfere with the completion of NAD^+^ transformation to NADH. In modern commercial kits, this is overcome by shifting an equilibrium through coupled reaction(s), usually including the reduction of formazan dyes, such as MTT. However, introduction of each additional coupled reaction makes the test prone to additional artifacts. Moreover, the artifacts are not possible to control when using commercial tests whose components are not disclosed. Furthermore, without additional procedures, the MTT-based tests, employing redox transformation of nicotinamide adenine dinucleotide, do not discriminate between the oxidized (NAD^+^) and reduced (NADH) forms of the dinucleotide. In particular, this is characteristic of the enzymatic studies mentioned in Supplementary Table 3. Although NADH and the redox ratio of NADH to NAD^+^ are important metabolic indicators, additional to NAD^+^ alone, the different chemical stability of NADH and NAD^+^ poses challenges for simultaneous quantification of both indicators in the same blood extract in clinical settings.

According to some estimates, the blood levels of NADH are 5–10 times lower than those of NAD^+^ (50), and thus should not contribute more than 20% to the total NAD pool, comprising both the oxidized and reduced dinucleotide. However, a two-fold variation in the range of the NAD pool, determined by the MTT-employing enzymatic tests (Supplementary Table 3), agrees with the notion that such tests are prone to various artifacts (32, 44, 45, 51). More importantly, comparison of the MTT-based tests relies on the arbitrary reaction time rather than the end-point titration, used in our FDH-based assay. In this case, if the sample composition differs, e.g., due to the various prescription drugs in different patients, the drugs may affect the MTT reduction, resulting in an artifactual change of the determined NAD pool. That is, the observed change may be related not to the NAD(H) content, but to the MTT reduction rate. Ascorbic acid, vitamin A, cellular sulfhydryl-containing compounds, such as reduced glutathione and coenzyme A, are known to reduce MTT to formazan (52–55). Varied content of these and other compounds in blood of different patients would thus contribute to the NAD-independent differences in the MTT-reduction. As an example, the MTT-based NADH assay in human erythrocytes shows diurnal oscillations, but they are coupled to changes in the cellular redox metabolism, including the changed expression of a highly abundant thiol-comprising redox enzyme, peroxiredoxin (56). These diurnal changes in the cellular redox metabolism raise questions regarding the nature of the MTT-detected changes, which do not necessarily reflect the NAD content. In case of the FDH reaction, the inhibitors or activators may also affect the reaction rate. However, in contrast to the MTT-based assay, the assay employing FDH is not dependent on the reaction rate or time, but based on the end-point titration of NADH itself, characterizing the complete transformation of NAD^+^ to NADH. Therefore, significant effects of different drugs prescribed to patients on the FDH-based NAD^+^ assay are not expected.

Apart from the high resistance to the artifacts discussed above, sensitivity of our fluorometric NAD^+^ assay corresponds to the detection limit of 5 pmoles NAD^+^ per 50 uL sample, or 100 nM NAD^+^, that is better than the detection limit of 400 nM NAD^+^, reported by Abcam^1^ or Biovision^2^ for the MTT-based assays.

It is worth noting that the tissue NAD^+^ or total NAD content may be subject to circadian changes, although the oscillation periods do not coincide in different studies (57–59). Probably, this poor coincidence is related to the fact that even in the liver tissue, where the NAD^+^ oscillation amplitudes are high, they are mostly within the standard errors of the NAD assays. Nevertheless, to exclude additional source of variations, the blood samples for our analyses are taken in the morning. Using the FDH-based test to detect potential circadian changes in NAD^+^ content in human blood may open new perspectives for chronobiological implications in medicine.

Decreased NAD^+^ content in demyelinating neuropathies is known from cellular and animal models of the diseases. For instance, rapid NAD^+^ drop upon Wallerian (injury-induced) degeneration of axons (29, 60), neurites (61) and in dissected nerves (62) suggests diminished NAD^+^ level in Charcot-Marie-Tooth disease and other peripheric neuropathies. Thus, our finding of decreased NAD^+^ levels in patients with demyelinating neuropathies is in good accordance with the model studies. Interestingly, the NAD pool is depleted in neurons exposed to toxic prion proteins, or in models of protein misfolding in Alzheimer’s and Parkinson’s diseases (63). Reduced level of total NAD in the brain tissue is observed in a mouse model of cerebellar ataxia (64), but the depletion does not occur in astrocytes or brain homogenates of scrapie-infected mice (63).

Similarly, decreased content of total NAD in myocardium is shown in mouse models of dilated cardiomyopathy (65) and failing heart (66). These model studies are in good accord with our finding of decreased levels of NAD^+^ in the whole human blood of cardiological patients (Figure 1). As mentioned in Introduction, the old (75–101 years) patients hospitalized for decompensated heart failure have a lower level of total NAD, compared to the younger (19–68 years) controls (11). In view of the published results on decreased NAD pool in cardiological pathologies, our finding of decreased NAD^+^ in the blood of cardiological patients suggests an impairment in NAD biosynthesis rather than increased NADH/NAD^+^ ratio in these patients. However, the abovementioned comparison of the MTT-based quantification of total NAD employs the cardiological patients and control subjects of different ages. Hence, further studies are needed to decipher the molecular mechanisms underlying decreases in NAD^+^ upon the cardio- and neuropathologies.

Thus, to the best of our knowledge, declines in the blood levels of NAD^+^ in the neurological and cardiological patients, shown in our work (Figure 1 and Supplementary Figure 2), have not been demonstrated before, although decreased NAD levels are known in healthy aging (16–18). Our data support this finding, as we also observe a slight decline in the blood NAD^+^ content in healthy subjects with age (Supplementary Figure 3). For the future studies it is important to note, however, that this and other correlations may be changed in different cohorts, manifesting certain specificity of pathophysiological changes in patients (Supplementary Figure 3 and Supplementary Table 2). Owing to this, the discriminating power of NAD^+^ as a clinical indicator of specific pathologies or as a risk factor of their development may be increased by taking into account potential associations of the NAD^+^ levels with other clinically relevant parameters (Figure 2 and Table 2). Further studies in these directions may be performed using the FDH-based NAD^+^ assay, developed in the current work. Remarkably, the associations between perturbed levels of NAD^+^ and creatinine, observed in our study (Figure 2), are also known in animals with acute and chronic kidney disease (67) and in patients with pellagra (68).

Supplementation with vitamin B3 (NAD precursor) to subjects under increased risks of neurological, cardiological and other disorders is currently considered as an efficient therapeutic strategy (69). Our easy NAD^+^ test may be applied to reveal the best therapies to increase the NAD^+^ level for protection from the age-related pathologies.

As a result, the proposed FDH-based assay of NAD^+^ in human blood may be useful for a more precise diagnosis of different pathologies and associated risks.

## Data Availability Statement

The raw data supporting the conclusions of this article will be made available by the authors, without undue reservation.

## Ethics Statement

The studies involving human participants were reviewed and approved by Independent Ethics Committee of M.F. Vladimirsky Moscow Regional Research and Clinical Institute. The patients/participants provided their written informed consent to participate in this study.

## Author Contributions

NB and AA performed the NAD^+^ measurement and quantification. NB and OS provided the blood samples and medical histories analyses. LS purified the recombinant FDH. VT supervised the FDH production. LZ, AP, and VB analyzed the results. VB and AP wrote the manuscript draft. VB, AA, and AT edited the manuscript. All authors read and agreed on the submitted version of the manuscript.

## Conflict of Interest

The authors declare that the research was conducted in the absence of any commercial or financial relationships that could be construed as a potential conflict of interest.

## Publisher’s Note

All claims expressed in this article are solely those of the authors and do not necessarily represent those of their affiliated organizations, or those of the publisher, the editors and the reviewers. Any product that may be evaluated in this article, or claim that may be made by its manufacturer, is not guaranteed or endorsed by the publisher.

## Footnotes

1
https://www.abcam.com/nadnadh-assay-kit-colorimetric-ab65348.html

2
https://www.biovision.com/nad-nadh-quantitation-colorimetric-kit.html
