# Supplementary material for: Efficient Assay and Marker Significance of NAD+ in Human Blood
Source: Front Med (Lausanne). 2022 May 19;9:886485. doi: 10.3389/fmed.2022.886485 (PMC9162244; doi:10.3389/fmed.2022.886485)
Supplement: Supplementary file 1 [file Data_Sheet_1.pdf]

Supplementary Material

1 Supplementary Figures

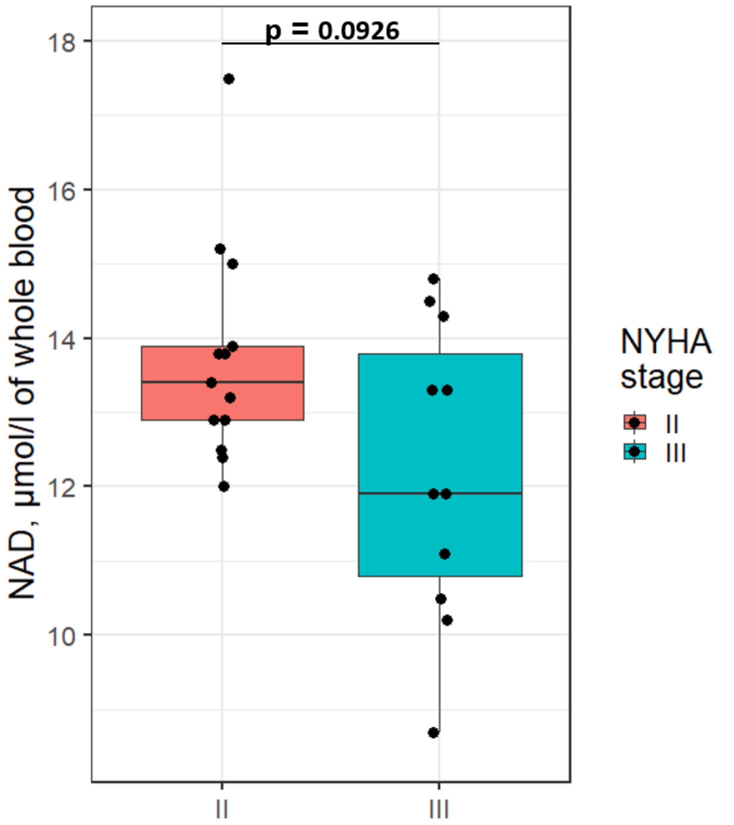

NYHA stage	min	median	max	age	count
II	12	13,4	18	42-83	13
III	8,7	11,9	15	52-80	11

Supplementary Figure S1. Differences in the concentration of NAD$^+$ in the whole blood of cardiological patients with the NYHA heart failure stages II and III. The samples exclude the outliers. The sample parameters are given in the table below the graph.

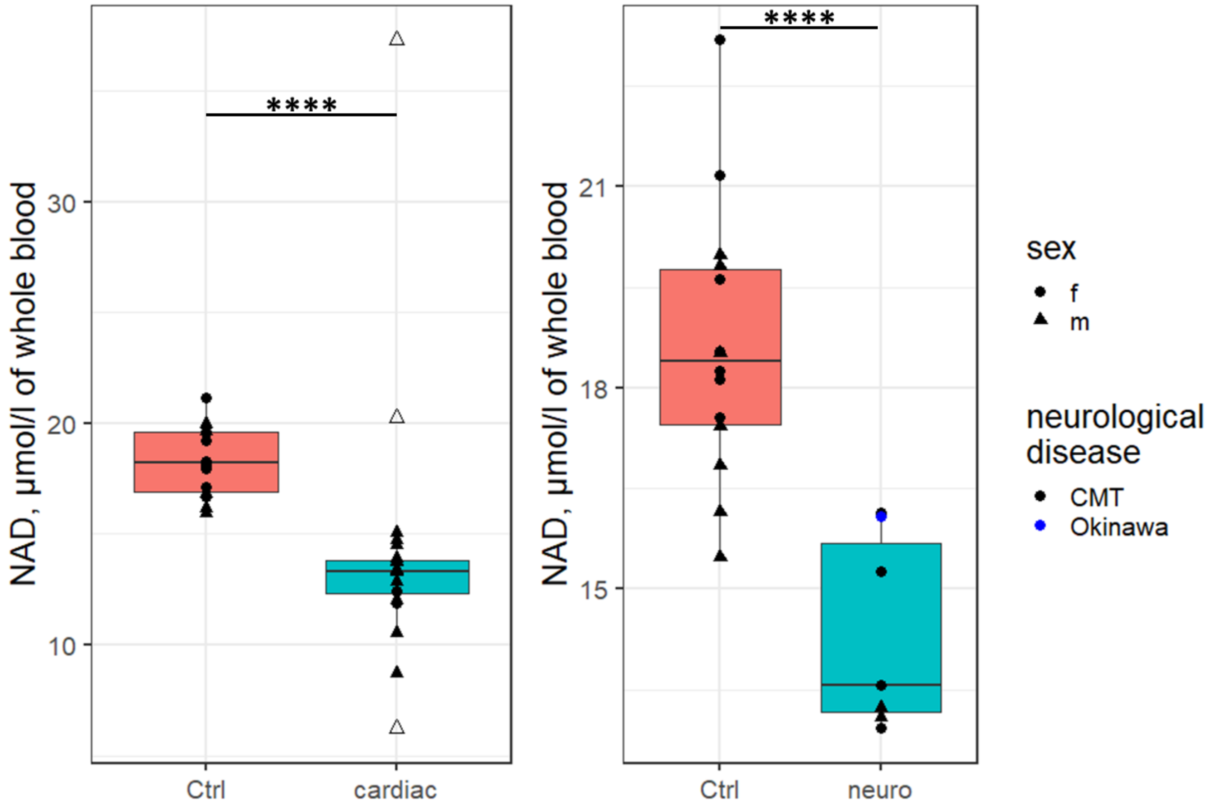

group	min	median	max	age	count
Ctrl	15,95	18,197	21,162	44-70	14
cardiac	8,7 (6,22)	13,325 (13,333)	15,035 (37,317)	42-68	16 (19)

group	min	median	max	age	count
Ctrl	15,475	18,393	23,194	25-48	14
neuro	12,92	13,575	16,133	27-55	7

Supplementary Figure S2. Differences in the concentration of NAD$^+$ in the whole blood of cardiological (left) and neurological (right) patients *vs* healthy volunteers of the respective age. Statistical significance on the graph corresponds to the sample excluding the outliers. The three NAD$^+$ outliers in the cardiological patients of the indicated age are shown as empty symbols on the left graph. The corresponding sample parameters, given in the table below the graph, are shown in parenthesis. The difference of the sample including the outliers, from the control one is characterized by p = 3,354e-05. **** – p < 0.0001.

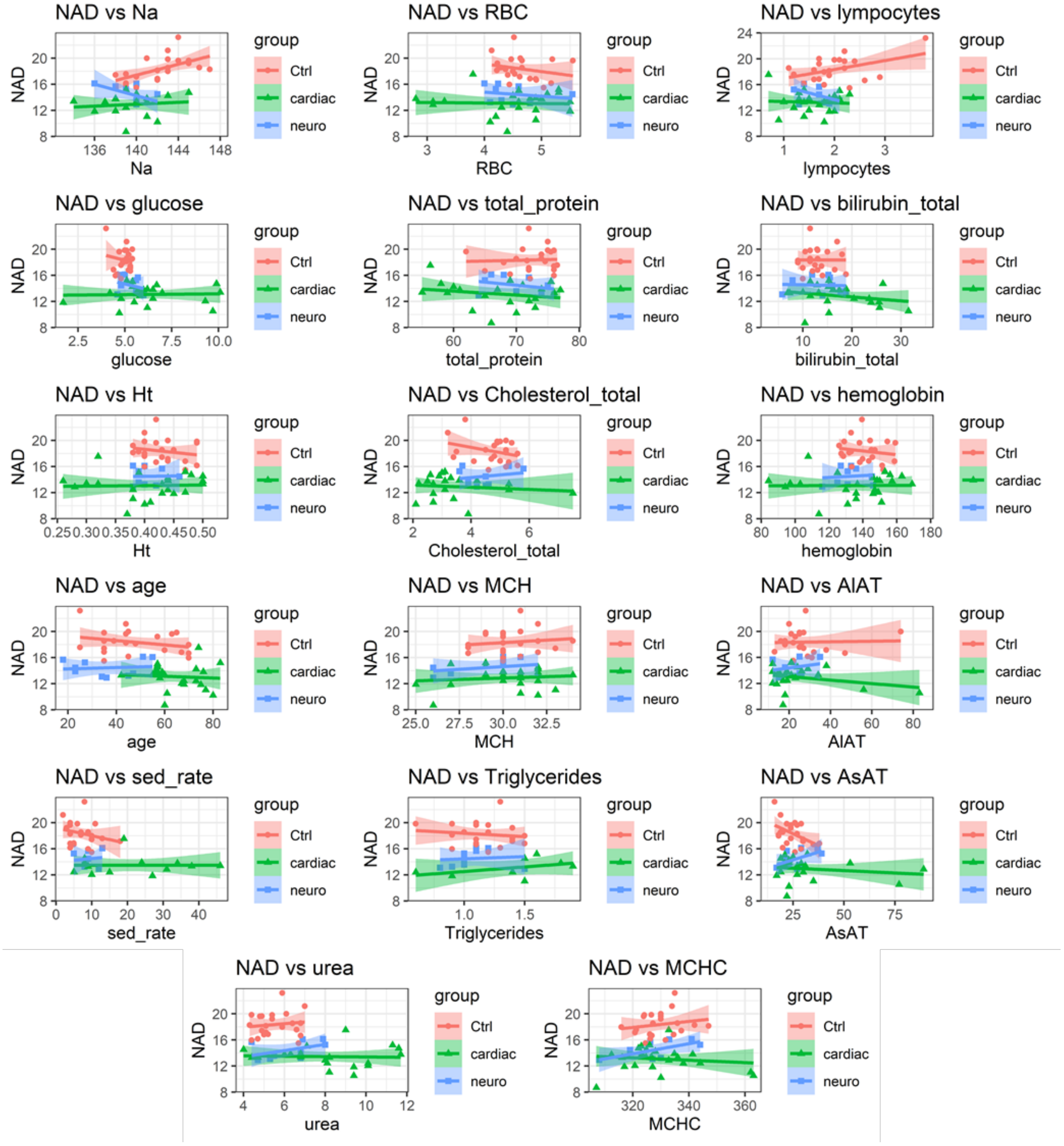

Supplementary Figure S3. Correlations of the NAD^+ content with other parameters of the blood.

2 Supplementary Tables

Supplementary Table S1. Comparison of the parameters of the clinical and biochemical blood analyses, available in studied cohorts. Medians with minimum (Min) and maximum (Max) values of the parameters including 4 outliers in cardiac patients are shown along with *p* values of statistical significances of the group differences, estimated by ANOVA, followed by the multiple group comparison using the post-hoc Tuckey's test. For NAD^{+} levels, the values in parenthesis correspond to those excluding the 4 outliers in cardiological patients.

Parameter	Group	Min	Median	Max	ANOVA, *p*	Multiple group comparisons, *p*	
						***vs* cardiac**	***vs* neuro**
	Ctrl	25.0	44.5	70.0		4.2E-05	0.03
	cardiac	42.0	63.5	83.0			1.2E-07
Age	neuro	18.0	35.0	55.0	3.0E-08		
						(2.36E-13)	(1.1E-06)
	Ctrl	15.5	18.2	23.2		0.003	0.039
		(8.7)	(13.3)	(17.5)			(0.11)
	cardiac	6.2	13.4	37.3	(2.4E-13)		1.0
NAD^{+}, μmol/l of whole blood	neuro	12.9	14.2	16.1	0.003		
	Ctrl	126.0	139.7	159.2		0.48	0.48
	cardiac	84.3	141.4	177.0			0.94
Hemoglobin	neuro	116	131	146	0.4		
	Ctrl	0.38	0.43	0.49		0.45	0.55
	cardiac	0.26	0.43	0.53			0.98
Hematocrit (Ht)	neuro	0.38	0.40	0.46	0.4		
	Ctrl	4.13	4.58	5.55		0.65	0.78
	cardiac	2.8	4.6	5.5			1.0
RBC	neuro	4.00	4.30	5.54	0.6		
	Ctrl	86	92	101		0.88	0.16
	cardiac	78	92	103			0.27
MCV	neuro	82	87	97	0.2		
	Ctrl	28	30	34		0.96	0.93
	cardiac	25	31	34			0.84
MCH	neuro	26	30	32	0.8		
	Ctrl	315.9	328.6	347.0		0.92	0.92
	cardiac	307.0	325.4	363.0			1.0
MCHC	neuro	308	327	344	0.9		
Lymphocytes	Ctrl	1.1	1.9	3.8	0.02	0.02	0.16

	cardiac	0.7	1.5	3.1			0.99
	neuro	1.2	1.7	2.1			
Sedimentation rate (sed_rate)	Ctrl	2.0	6.5	18.0		1.1E-05	0.64
	cardiac	5	19	46			0.004
	neuro	5	9	13	1.5E-05		
Bilirubin_total	Ctrl	9.0	12.7	18.8		2.3E-05	0.25
	cardiac	6.2	16.1	44.2			0.09
	neuro	5.8	15.2	18.8	4.1E-05		
Total_protein	Ctrl	62.0	73.1	76.5		0.006	0.61
	cardiac	43.0	69.0	78.0			0.31
	neuro	64.0	70.0	76.0	0.008		
Urea	Ctrl	4.3	5.3	7.0		2.0E-05	0.51
	cardiac	4.0	8.1	11.7			0.02
	neuro	4.4	6.8	8.0	2.9E-05		
Creatinine	Ctrl	58.0	83.5	96.0		0.0002	0.96
	cardiac	74.0	99.4	178.0			0.01
	neuro	73.0	85.0	92.0	0.0001		
Glucose	Ctrl	4.0	5.1	5.4		0.003	0.78
	cardiac	1.7	6.0	10.1			0.13
	neuro	4.6	5.6	6.0	0.003		
K^+	Ctrl	4.2	4.5	4.9		0.98	0.40
	cardiac	3.7	4.5	6.0			0.32
	neuro	4.1	4.1	5.1	0.33		
Na^+	Ctrl	138	143	147		0.013	0.03
	cardiac	134	139	149			0.92
	neuro	136	140	142	0.006		
Alanine aminotransferase (AlAT)	Ctrl	13	25	74		0.61	1.0
	cardiac	12	21	838			0.68
	neuro	12	23	35	0.55		
Aspartate aminotransferase (AsAT)	Ctrl	16	24	38		0.16	0.98
	cardiac	17	26	237			0.46
	neuro	17	27	39	0.15		
Cholesterol_total	Ctrl	3.2	4.9	5.6		0.0001	0.77
	cardiac	2.1	3.1	7.5			0.03
	neuro	3.6	4.5	5.8	0.0001		

	Ctrl	0.6	1.1	1.5		0.17	0.76
	cardiac	0.6	1.5	1.9			0.09
Triglycerides	neuro	0.8	1.0	1.5	0.09		

Supplementary Table S2. Correlations of NAD$^+$ content with the age and parameters of the clinical and biochemical blood analyses, available in studied cohorts. Only NAD$^+$ correlation with Na$^+$ in the control group is significant after multiple testing adjustment (p adjusted = 0.0331), marked in green. Grey marks the raw values of $p \leq 0.1$. The four NAD$^+$ outliers are excluded from the correlation analysis.

**NAD$^+$**	**Spearman correlation r**			***p* values**		
**vs**	**control**	**cardio**	**neuro**	**control**	**cardio**	**neuro**
**Age**	-0.2250	-0.2377	0.1216	0.3142	0.2634	0.7379
**Creatinine**	-0.2337	0.0775	0.0084	0.2952	0.7190	0.9830
**MCV**	0.0159	0.1503	0.3698	0.9440	0.4832	0.3274
**K$^+$**	0.4128	-0.1067	0.0183	0.0562	0.6451	0.9628
**Na$^+$**	0.6306	0.2106	-0.5660	0.0017	0.3867	0.1122
**RBC**	-0.2728	0.0606	-0.4000	0.2193	0.7784	0.2861
**Lymphocytes**	0.1988	0.0892	-0.3109	0.3751	0.6857	0.4154
**Glucose**	0.0925	0.1209	-0.2204	0.6821	0.5919	0.5688
**Total protein**	0.0147	-0.1507	-0.2000	0.9481	0.5142	0.6059
**Bilirubin total**	-0.0159	-0.1834	-0.0667	0.9441	0.4023	0.8647
**Hematocrit**	-0.1819	0.1916	0.0084	0.4178	0.3697	0.9828
**Cholesterol total**	-0.1899	-0.0724	0.0251	0.3973	0.7616	0.9489
**Hemoglobin**	-0.1807	0.1566	0.1088	0.4209	0.4650	0.7806
**Age**	-0.2250	-0.2377	0.1216	0.3142	0.2634	0.7379
**MCH**	0.1396	-0.0596	0.1368	0.5354	0.7870	0.7256
**AlAT**	-0.1720	-0.0446	0.2259	0.4441	0.8400	0.5588
**Sedimentation rate**	-0.2777	-0.0055	0.2797	0.2108	0.9858	0.4660
**Triglycerides**	-0.2086	0.5509	0.3405	0.3642	0.1570	0.3699
**AsAT**	-0.4011	-0.1596	0.5000	0.0643	0.4670	0.1705
**Urea**	0.1312	-0.1069	0.5356	0.5605	0.6537	0.1373
**MCHC**	0.1860	-0.1113	0.6000	0.4073	0.6045	0.0876

Supplementary Table S3. Comparison of the NAD^+ concentration in whole blood of healthy volunteers, measured in this study using FDH assay, with published data. The values of NAD^+ in the whole blood are presented as mean ± SEM or as intervals of means obtained in similar studies. * - The MTT-employed assay does not differentiate the reduced and oxidized form of nicotinamide adenine dinucleotide (NAD)

Analytical technique	NAD^+, µM	References
Fluorometric enzymatic assay with formate dehydrogenase	18 ± 2	This study
Liquid chromatography-coupled mass spectrometry	17-27	[1-3]
Nuclear magnetic resonance	16 ± 1	[4]
Colorimetric enzymatic assay with alcohol dehydrogenase and MTT*	23-41	[5-7]

References:

1. Airhart, S. E., Shireman, L. M., Risler, L. J., Anderson, G. D., Nagana Gowda, G. A., Raftery, D., Tian, R., Shen, D. D. & O'Brien, K. D. (2017) An open-label, non-randomized study of the pharmacokinetics of the nutritional supplement nicotinamide riboside (NR) and its effects on blood NAD+ levels in healthy volunteers, *PloS one.* **12**, e0186459.

2. Giner, M. P., Christen, S., Bartova, S., Makarov, M. V., Migaud, M. E., Canto, C. & Moco, S. (2021) A Method to Monitor the NAD(+) Metabolome-From Mechanistic to Clinical Applications, *International journal of molecular sciences.* **22**.

3. Dellinger, R. W., Santos, S. R., Morris, M., Evans, M., Alminana, D., Guarente, L. & Marcotulli, E. (2017) Repeat dose NRPT (nicotinamide riboside and pterostilbene) increases NAD(+) levels in humans safely and sustainably: a randomized, double-blind, placebo-controlled study, *NPJ Aging Mech Dis.* **3**, 17.

4. Nagana Gowda, G. A. & Raftery, D. (2017) Whole Blood Metabolomics by (1)H NMR Spectroscopy Provides a New Opportunity To Evaluate Coenzymes and Antioxidants, *Analytical chemistry.* **89**, 4620-4627.

5. Creeke, P. I., Dibari, F., Cheung, E., van den Briel, T., Kyroussis, E. & Seal, A. J. (2007) Whole blood NAD and NADP concentrations are not depressed in subjects with clinical pellagra, *The Journal of nutrition.* **137**, 2013-7.

6. Breton, M., Costemale-Lacoste, J. F., Li, Z., Lafuente-Lafuente, C., Belmin, J. & Mericskay, M. (2020) Blood NAD levels are reduced in very old patients hospitalized for heart failure, *Experimental gerontology.* **139**, 111051.

7. Shibata, K. & Tanaka, K. (1986) Simple measurement of blood NADP and blood levels of NAD and NADP in humans, *Agricultural and Biological Chemistry.* **50**, 2941-2.
